# Oxidative stress induces club cell proliferation and pulmonary fibrosis in Atp8b1 mutant mice

**DOI:** 10.18632/aging.101742

**Published:** 2019-01-13

**Authors:** Jutaro Fukumoto, Joseph Leung, Ruan Cox, Alexander Czachor, Prasanna Tamarapu Parthasarathy, Venu Lagishetty, Maria Mandry, Nima Hosseinian, Priyanshi Patel, Brittany Perry, Mason T. Breitzig, Matthew Alleyn, Athena Failla, Young Cho, Andrew J. Cooke, Lakshmi Galam, Ramani Soundararajan, Nirmal Sharma, Richard F. Lockey, Narasaiah Kolliputi

**Affiliations:** 1 Division of Allergy and Immunology, Department of Internal Medicine, Morsani College of Medicine, University of South Florida, Tampa, FL 33612, USA; 2 Department of Molecular Medicine, Morsani College of Medicine, University of South Florida, Tampa, FL 33612, USA; 3 Advanced Lung Diseases & Lung Transplantation, Internal Medicine, Morsani College of Medicine, University of South Florida, Tampa, FL 33612, USA

**Keywords:** hyperoxia, Atp8b1, idiopathic pulmonary fibrosis (IPF), oxidative stress, club cells

## Abstract

Atp8b1 (ATPase, aminophospholipid transporter, class I, type 8B, member 1) is a cardiolipin transporter in the apical membrane of lung epithelial cells. While the role of Atp8b1 in pneumonia-induced acute lung injury (ALI) has been well studied, its potential role in oxidative stress-induced ALI is poorly understood. We herein show that Atp8b1^G308V/G308V^ mice under hyperoxic conditions display exacerbated cell apoptosis at alveolar epithelium and aberrant proliferation of club cells at bronchiolar epithelium. This hyperoxia-induced ambivalent response in Atp8b1^G308V/G308V^ lungs was followed by patchy distribution of non-uniform interstitial fibrosis at late recovery phase under normoxia. Since this club cell abnormality is commonly observed between Atp8b1^G308V/G308V^ lungs under hyperoxic conditions and IPF lungs, we characterized this mouse fibrosis model focusing on club cells. Intriguingly, subcellular morphological analysis of IPF lungs, using transmission electron microscopy (TEM), revealed that metaplastic bronchiolar epithelial cells in fibrotic lesions and deformed type II alveolar epithelial cells (AECs) in alveoli with mild fibrosis, have common morphological features including cytoplasmic vacuolation and dysmorphic lamellar bodies. In conclusion, the combination of Atp8b1 mutation and hyperoxic insult serves as a novel platform to study unfocused role of club cells in IPF.

## Introduction

Idiopathic pulmonary fibrosis (IPF) is a chronic, progressive, fibrotic lung disease of unknown etiology [1]. The median survival of this fatal disease after diagnosis is approximately 3 years and the outcome is largely unaffected by current therapies [2]. The biggest challenge that has hindered the prevention and treatment of IPF is its heterogeneous nature: heterogeneity in etiology, histopathology, and clinical course. Indeed, the current, widely used bleomycin-induced mouse model of pulmonary fibrosis has helped researchers and clinicians understand IPF pathophysiology to a large extent as it mimics many pathological aspects of IPF [3]. However, the events that occur in the early stages of IPF have not been well investigated in the belomycin model. In this model, fibrosis is preceded by robust inflammation, which is inconsistent with the current consensus that IPF is not triggered by inflammation.

Recent advances in molecular and cellular biology have led to a consensus that continually damaged type II alveolar epithelial cells (AECs) serve as a trigger for IPF [4]. Compared to type II AECs, the role for club cells in IPF pathology is unclear and has been largely deemphasized until recently [5,6]. The relative difficulty of obtaining a pure population of primary club cells, compared to type II AECs, has also hindered our understanding of club cell behavior in IPF development.

Alveolar bronchiolization, a process of proliferation and alveolar migration of club cells and other bronchiolar epithelial cell types, has been documented since the 1970s [7]. Its potential relevance to pulmonary fibrosis has been examined in IPF as well as animal models of lung fibrosis [6,8,9]. A systematic lineage tracing approach supports the concept that migration of club cells is an inherent repair system [10], however a recent report by Akram et al. suggests that the migratory club cells contribute to IPF progression by promoting lung epithelial cell death [5]. They revealed that in IPF lungs TRAIL-expressing club cells, which are suspected to induce type II alveolar epithelial cell apoptosis, were spotted within the affected alveolar epithelium in areas of established fibrosis. Thus, the involvement and the role of club cells in IPF pathogenesis need further investigation.

Atp8b1 (ATPase, aminophospholipid transporter, class I, type 8B, member 1), encoded by ATP8B1/ FIC1/PFIC1, is a membrane-bound protein. Specific mutations in ATP8B1 gene were originally identified as responsible for Byler’s disease: a genetic liver disorder characterized by progressive intrahepatic cholestasis that ultimately leads to liver cirrhosis [11]. Revealed and highlighted by Ray et al. afterward, was a role for Atp8b1 in the lung as a cardiolipin transporter in type II AECs. With this characteristic, Atp8b1 lowers the elevated cardiolipin level in the airspace which is caused by acute lung injury (ALI) [12]. The cardiolipin clearance function of Atp8b1 cushions the rapid increase of surface tension in the lung, maintains lung function, and counteracts epithelial apoptosis in ALI [12]. While the elevation of cardiolipin in lung airspace compromises the integrity of the lung, cardiolipin is a major lipid component of mitochondrial inner membrane and plays a dynamic role in maintaining the electron transport chain (ETC) and mediating mitochondria-dependent cell apoptosis [13,14]. Given such importance of cardiolipin in mitochondrial dynamics, we hypothesized that Atp8b1 loss-of-function causes loss-of-integrity in mitochondria under various mitochondria stressors including oxidative stress.

Hyperoxic insult, one of the most feasible method to inflict oxidative stress, preferentially oxidizes cardiolipin among other types of lipids in the cell [15], an event which prompts apoptosis [16]. Adamson et al. demonstrated, by using explants of mouse lung, that hyperoxia-induced lung damage, especially severe lung epithelial cell death, disturbs the normal reparative processes of the lung and promotes fibrotic reactions with minimal involvement of infiltrating immune cells [17]. In favor of the relationship between hyperoxia and lung fibrosis, hyperoxic insult has been used to phenocopy bronchopulmonary dysplasia (BPD), a chronic lung disease of the neonate which features extensive inflammatory and fibrotic changes in the airways and lung parenchyma [18]. Collectively, we hypothesized that hyperoxic insult would cause mitochondrial dysfunction-related cell death, which under particular conditions would trigger profibrotic responses in the lung.

Recent reports suggest that the combination of inherent epithelial dysfunction (genetic predisposition) and continual exposure to noxious stimuli (environmental factors) synergistically triggers IPF development [19]. In light of this concept as well as the aforementioned profile of Atp8b1 and hyperoxic insult, we hypothesized that the in vivo combination of loss-of-function mutation in ATP8B1 (genetic predisposition to epithelial cell dysfunction) and hyperoxia (environmental factors triggering mitochondria-mediated epithelial cell damage) synergistically (1) cause abnormal behaviors of lung epithelial cells including apoptosis and thereby (2) provokes delayed alveolar re-epithelialization accompanying aberrant fibrotic reactions in the lung.

## RESULTS

### Atp8b1^G308V/G308V^ mice under hyperoxic conditions display accelerated alveolar cell death and patchy proliferation of bronchiolar epithelial cells

**Figure 1:**
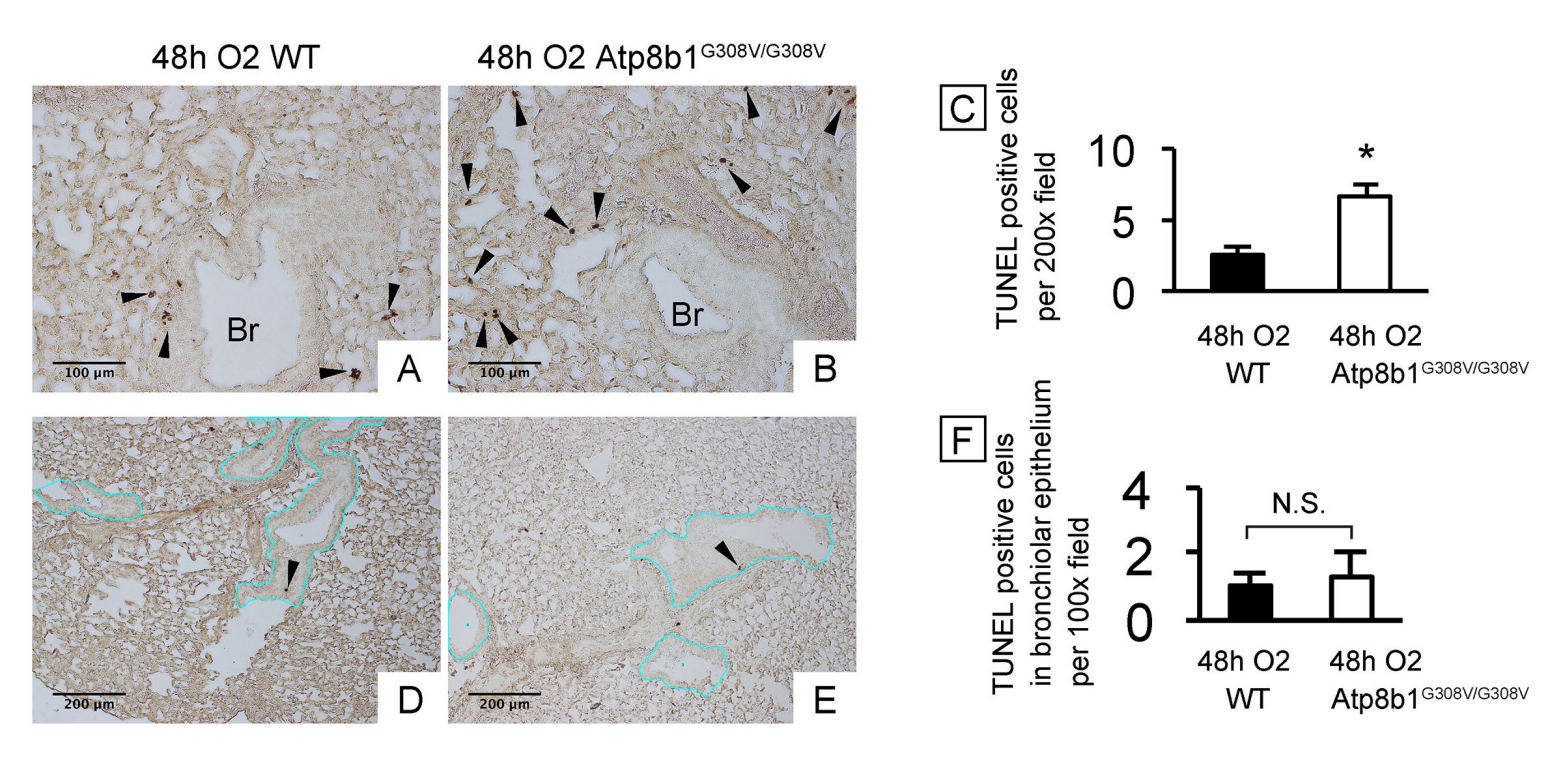
**Atp8b1^G308V/G308V^ mice under hyperoxic conditions display increased cell death in alveoli, but not in bronchiolar epithelium.** Wild-type (WT) and Atp8b1^G308V/G308V^ mice were exposed to 100% O_2_ for 48 hrs. Mice were euthanized and formaldehyde fixed paraffin-embedded lung sections were stained with terminal deoxynucleotidyl transferase dUTP nick end labeling (TUNEL). TUNEL-positive cells are denoted by arrowheads. (**A** & **B**) Representative photomicrographs focusing on bronchovascular bundles with surrounding alveoli. (**C**) Quantitative comparison between hyperoxic WT and Atp8b1^G308V/G308V^ mice (n=3 for each) regarding the total number of TUNEL positive cells per 100x field in the lung. The numbers of TUNEL-positive cells were determined in 7-8 randomly chosen 100x fields for each section. Means ± SE for each group is shown. *p < 0.05. (**D** & **E**) Representative photomicrographs of peripheral part of the lung with relatively small bronchioles. Basement membranes of bronchiolar epithelium are highlighted by blue lines. (**F**) Quantitative comparison between hyperoxic WT and Atp8b1^G308V/G308V^ mice (n=3 for each) regarding the number of TUNEL positive cells in bronchiolar epithelium. The number of TUNEL-positive cells in bronchiolar epithelium were determined in 7-8 randomly chosen 100x fields. Means ± SE is shown. *p < 0.05. Br: Bronchiolar lumen. Magnifications: (A & B) 200X; (D & E) 100X

Cardiolipin, an important component of the mitochondrial inner membrane, is essential for mitochondrial function as well as for the maintenance of the electric potential and integrity of mitochondrial membrane. Since Atp8b1 is a cardiolipin transporter in type II AECs, we speculated that loss-of-function of Atp8b1 would disturb cardiolipin homeostasis in type II AECs and thereby lower the apoptotic threshold under oxidative conditions. To determine if Atp8b1 deficiency accelerates morbidity of the lung under hyperoxic conditions, WT and Atp8b1^G308V/G308V^ mice were exposed to normoxia or hyperoxia for 48 hrs. Hyperoxia was chosen as a mitochondrial stressor and apoptosis inducer, because cardiolipin is a primary target of lipid oxidization in hyperoxic lung [15] and oxidized cardiolipin is a trigger for mitochondria-mediated cell apoptosis [16]. H&E-stained lung sections from normoxic Atp8b1^G308V/G308V^ mice did not show particular morphological phenotypes under light microscopy when compared to WT controls (data not shown). To assess the initial inflammatory and apoptotic response of Atp8b1^G308V/G308V^ mice to hyperoxic insult, lungs and bronchoalveolar lavage (BAL) fluid collected at 48 hrs of hyperoxia were evaluated. TUNEL staining on tissue sections from WT lungs spotted TUNEL-positive apoptotic cells in bronchiolar epithelium, alveoli, and other connective tissues including vascular structures (Fig. 1A). Atp8b1^G308V/G308V^ lungs displayed increased number of apoptotic cells in alveoli (Fig. 1B) as well as connective tissues near the bronchovascular bundles compared to WT controls. This increase in TUNEL positive cells in hyperoxic Atp8b1^G308V/G308V^ lungs was statistically significant (Fig. 1C). We surmise that this increase is, at least in part, due to the increased number of apoptotic type II AECs based on the observation that there was no statistically significant difference between hyperoxic WT and Atp8b1^G308V/G308V^ lungs regarding the number of TUNEL positive cells in bronchiolar epithelium (Fig. 1D-F; the basement membrane of bronchiolar epithelium is outlined by blue line). These results suggest that alveolar epithelial cells in Atp8b1^G308V/G308V^ mice are more vulnerable to oxidative stress-induced apoptosis compared to WT mice.

**Figure 2:**
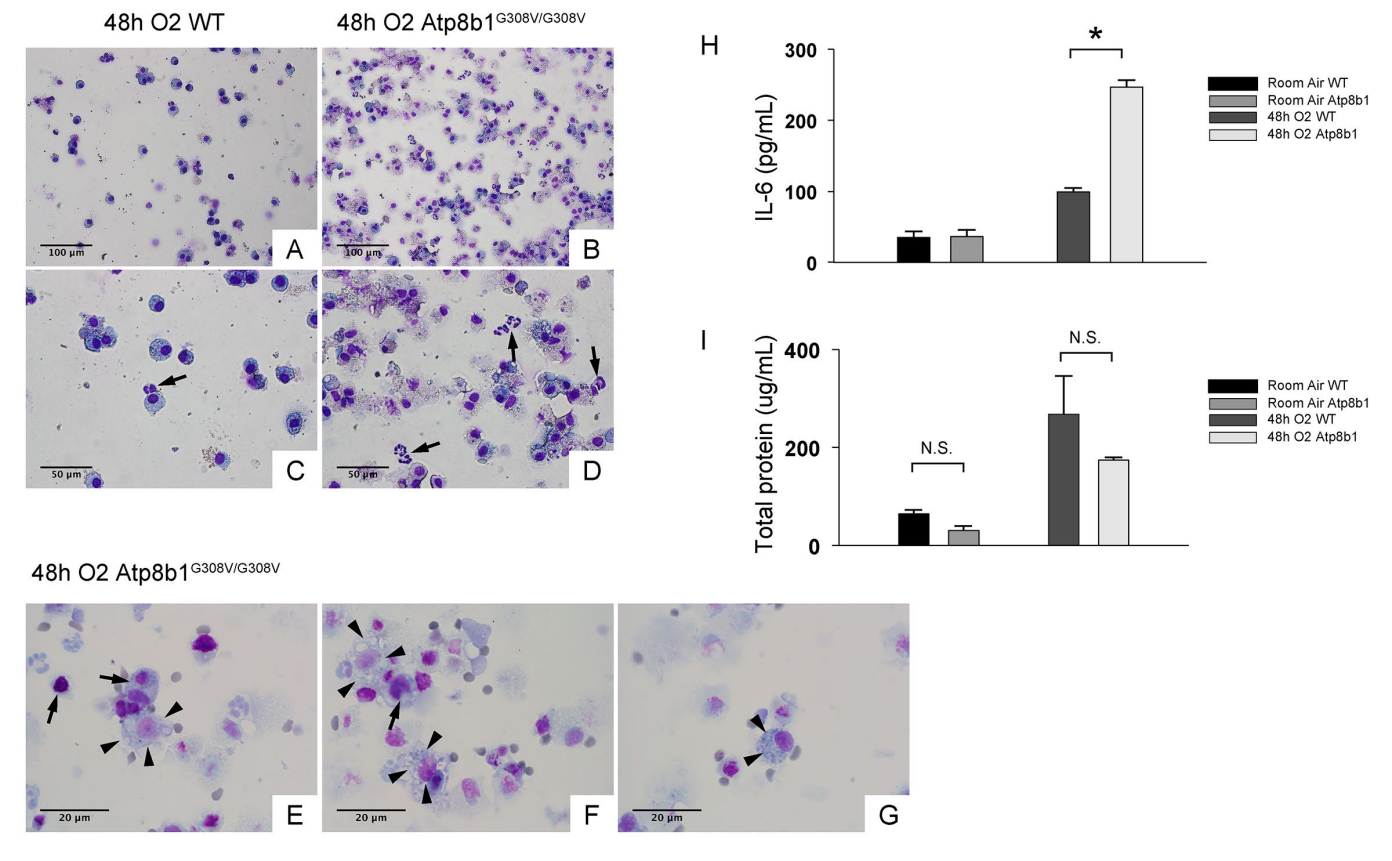
**Atp8b1^G308V/G308V^ mice under hyperoxic conditions display increased number of total cells in airspace compared to WT controls.** Representative photomicrographs of bronchoalveolar lavage fluid (BAL) cells retrieved from WT (**A** & **C**) and Atp8b1^G308V/G308V^ mice (n=3 for each) (**B, D** & **E-G**) following exposure to 100% O_2_ for 48 hrs. BAL fluid (BALF) cells were stained with Diff-Quik. Infiltrating neutrophils are indicated by arrows in Panel C & D. Highly vacuolated cells with weakly stained nucleus are encountered in airspace of hyperoxic Atp8b1^G308V/G308V^ mice (arrowheads in Panel E & F), which are morphologically distinct from surrounding cells that are considered to be macrophages (arrows in Panel E & F). Cells with eccentric nucleus and numerous cytoplasmic granules are occasionally encountered in hyperoxic Atp8b1^G308V/G308V^ mice, which are not morphologically similar to any immune cell types that are normally encountered in lung airspace (cell designated by arrowheads in Panel G). (**H** & **I**) Levels of IL-6 and total protein in BALF from WT and Atp8b1^G308V/G308V^ mice exposed to normoxia or 100% O_2_ for 48 hrs. IL-6 levels in BALF were measured by ELISA (n=3 for each group). Results are presented as Means ± SE. *p < 0.05. Magnifications: (A & B) 200X; (C & D) 400X; (E-G) 1000X. Data presented are representative of two independent experiments.

Increased lung permeability and immune cell infiltration are hallmarks of hyperoxia-induced lung injury. To determine if these inflammatory responses are enhanced by Atp8b1 deficiency, the cells in lung airspace were collected by bronchoalveolar lavage (BAL) and assessed for the number and types of the cell by microscopic observations. The majority of the cells in BAL fluid (BALF) from hyperoxic WT mice were round to oval in shape (Fig. 2C & D). They are considered to be alveolar macrophages because they do not display morphological features of other immune cell types that are typically recruited to damaged lungs such as neutrophils (arrow in Fig. 2C) or lymphocytes. In contrast, BALF cells from hyperoxic Atp8b1^G308V/G308V^ mice showed robust increase in the number of total cells compared to WT controls (Fig. 2B & D). Remarkably, unexpected cell types that are morphologically different from alveolar macrophage represented a large part of the BALF cells that had been retrieved from hyperoxic Atp8b1^G308V/G308V^ mice. These cells often displayed vacuolated cytoplasm and a big nucleolus (arrowheads in Fig. 2E & F). Additionally, a particular cell type reminiscent of club cells (long oval in shape, polarized nuclear location, and numerous cytoplasmic granules) was occasionally encountered (arrowheads in Fig. 2G). These results suggest the possibility that the increased number of cells in BAL fluid in hyperoxic Atp8b1^G308V/G308V^ mice is attributed to bronchiolar epithelial cells that have entered into airspace.

**Figure 3:**
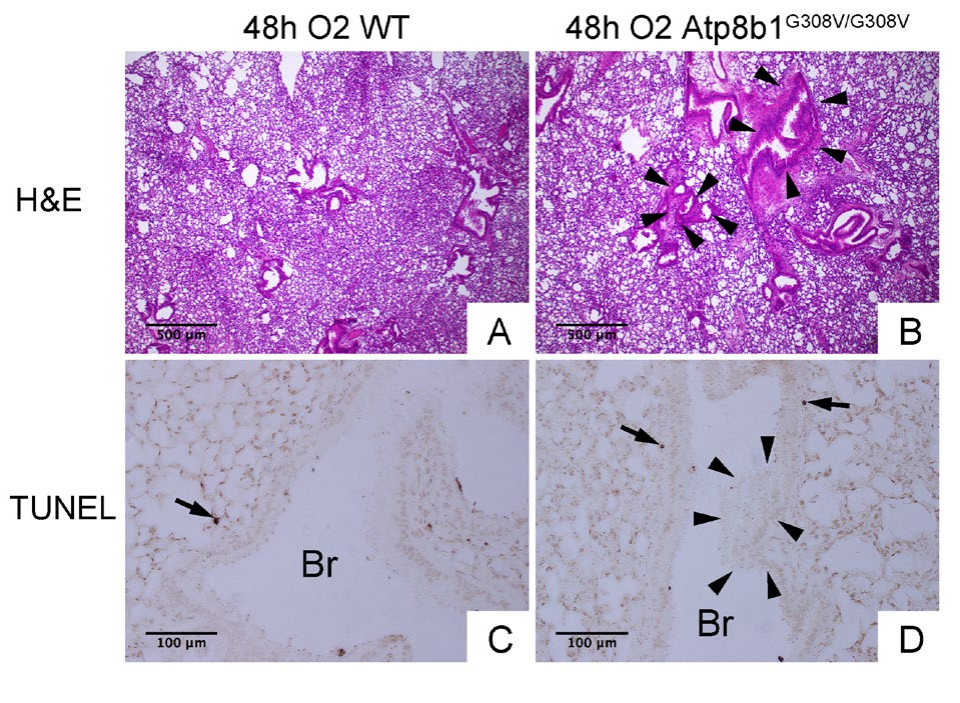
**Atp8b1^G308V/G308V^ mice under hyperoxic conditions display patchy thickening of bronchiolar epithelium with epithelial hypercellularity.** (**A** & **B**) Photomicrographs of H&E stained lung sections from WT and Atp8b1^G308V/G308V^ mice exposed to 100% O_2_ for 48 hrs (n=3 for each). (**C** & **D**) Photomicrographs of TUNEL stained lung sections from WT and Atp8b1^G308V/G308V^ mice exposed to 100% O_2_ for 48 hrs. Arrowheads denote thickened bronchiolar epithelium of hyperoxic Atp8b1^G308V/G308V^ lungs. TUNEL positive cells are denoted by arrows. Br: Bronchiolar lumen. Magnifications: (A & B) 100X; (C & D) 200X. Data presented are representative of two independent experiments.

**Figure 4:**
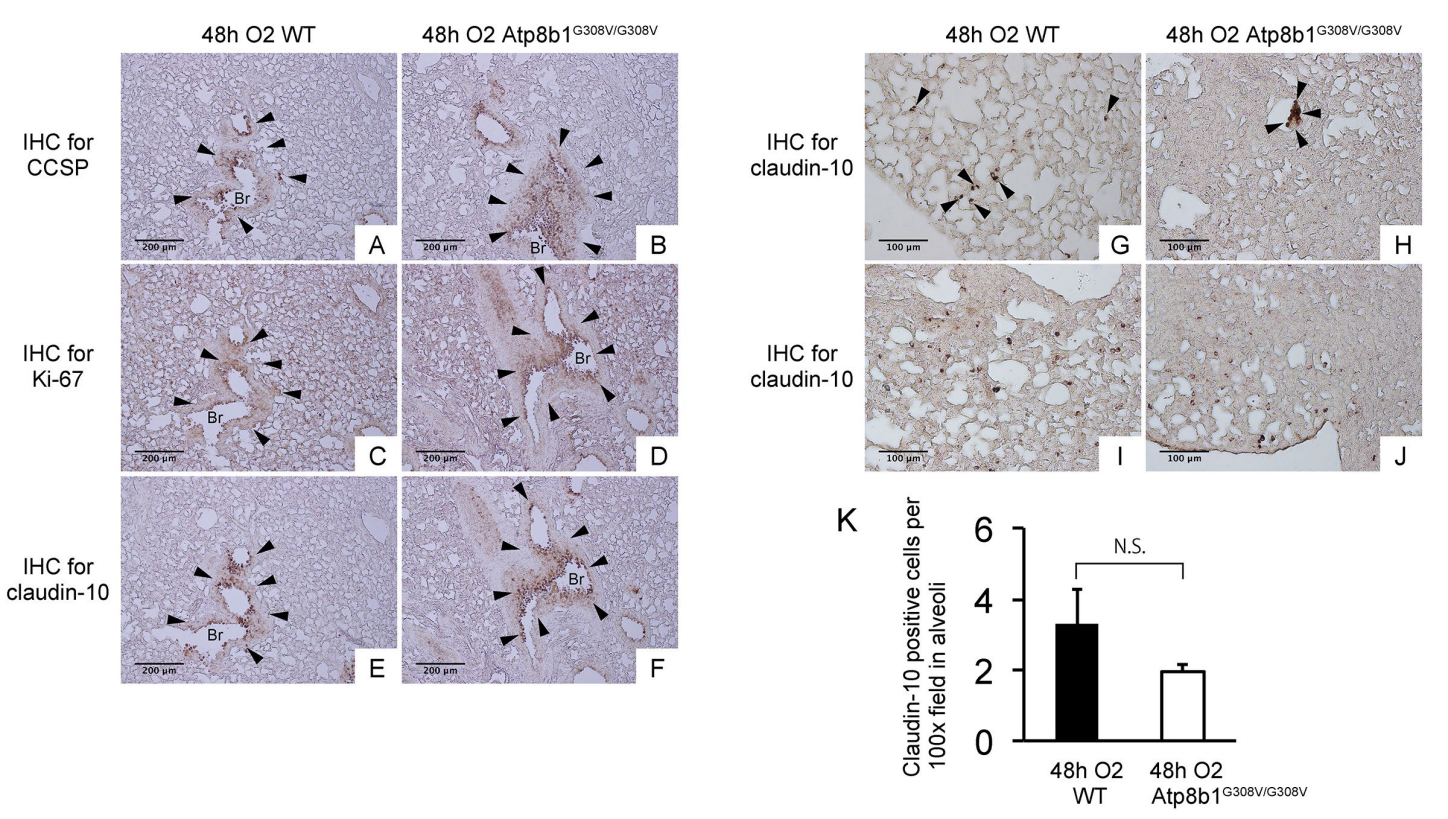
**Atp8b1^G308V/G308V^ mice under hyperoxic conditions display proliferation of claudin-10-positive club cells in thickened bronchiolar epithelium**. Paraffin-embedded lung sections from WT and Atp8b1^G308V/G308V mice^ exposed to 100% O_2_ for 48 hrs were subjected to immunohistochemical staining for club cell secretory protein (CCSP). (**A** & **B**), Ki-67 (**C** & **D**), and claudin-10 (**E**-**J**) (n=3 for each of WT and Atp8b1 mutant mice). Photomicrographs show representative images from either parabronchiolar (**A**-**F**) or alveolar regions (**G**-**J**) Arrowheads in Figure A-H designate positive cells for the respective markers. (**K**) claudin-10 positive cells per 100x field in alveoli were quantified in 10 randomized independent fields of 4 mice per each group. Means ± SE of the total number of claudin-10 positive cells for each group is shown. Br: Bronchiolar lumen. Magnifications: (A-F) 100X (G-J) 200X. Data presented are representative of two independent experiments.

Interleukin-6 (IL-6) is a representative inflammatory cytokine that protects against oxidative stress-induced cell damage. IL-6 level was significantly elevated in the airspace of hyperoxic Atp8b1^G308V/G308V^ mice compared to WT controls (Fig. 2H). On the other hand, there was no significant difference between hyperoxic WT and Atp8b1^G308V/G308V^ mice regarding total protein concentration in BAL fluid (Fig. 2I). These results indicate that Atp8b1 deficiency accelerates oxidative stress-induced IL-6 elevation in the airspace, but not lung permeability. The microscopic images obtained from H&E-stained lung tissue sections displayed a patchy thickening of bronchiolar epithelium in hyperoxic Atp8b1^G308V/G308V^ lungs (arrowheads in Fig. 3B). To test if this bronchiolar epithelial hypercellularity seen in hyperoxic Atp8b1^G308V/G308V^ lungs reflects an accumulation of apoptotic bronchiolar epithelial cells that have not been processed by phagocytes such as alveolar macrophages, TUNEL staining was performed. The results show that most of the cells, in the thickened bronchiolar epithelium of Atp8b1^G308V/G308V^ lungs, are negative for TUNEL (arrowheads in Fig. 3D). Note there are TUNEL-positive cells nearby around the basement membrane (arrows in Fig. 3D). Therefore, we hypothesized that the bronchiolar epithelial hypercellularity observed in hyperoxic Atp8b1^G308V/G308V^ mice represents emergence and proliferation of an oxidative stress-resistant cell type that serves as a protection against lung epithelial damage. Since club cells are a well-known lung-specific progenitor cell type that resides at bronchiolar epithelium, we also hypothesized that a certain subpopulation of club cell type proactively proliferates under hyperoxic conditions in Atp8b1^G308V/G308V^ lungs. To corroborate this hypothesis, serially cut lung tissue sections from WT and Atp8b1^G308V/G308V^ mice exposed to 48 hrs of hyperoxia were immunohistochemically labeled for Ki-67 (a proliferation marker), club cell secretory protein (CCSP) (a club cell marker), and claudin-10 (a second club cell marker). The results show that a large portion of bronchiolar epithelial cells in thickened bronchiolar epithelium, observed in Atp8b1^G308V/G308V^ mice at 48 hrs of hyperoxia, are Ki-67-positive, CCSP-positive and claudin-10-positive (Fig. 4B, D & F). These results indicate that alveolar epithelial cell death under hyperoxic conditions is accompanied by proliferation of oxidative stress-resistant club cells in Atp8b1^G308V/G308V^ mice. Proliferative club cells are also seen in bronchiolar epithelium in WT mice under hyperoxia to a lesser extent compared to Atp8b1^G308V/G308V^ mice (Fig. 4A, C & E). These data suggest that specific subpopulation of club cells is resistant to the apoptosis induced by oxidative stress and that reservoir of this population is increased in Atp8b1^G308V/G308V^ mice. The close observation of claudin-10-positive cells in Atp8b1^G308V/G308V^ lungs revealed that cells positive for claudin-10 were spotted as a cell cluster in collapsed alveoli (Fig. 4H) or as a single colony in relatively unaffected lesions (Fig. 4J). Meanwhile, most of the claudin-10-positive cells in WT lungs were found scattered in relatively intact alveoli (Fig. 4G & I). There was no difference between hyperoxic WT and Atp8b1^G308V/G308V^ lungs regarding the number of claudin-10-positive cells in alveoli (Fig. 4K). These results suggest that excessive proliferation of claudin-10-positive club cells at bronchiolar epithelium and their abnormal behavior in alveoli impede the restoration of normal alveolar architecture in hyperoxic Atp8b1^G308V/G308V^ lungs.

Collectively, these results led us to a hypothesis that Atp8b1^G308V/G308V^ mice exhibit accelerated lung epithelial cell apoptosis under hyperoxic insult, which provokes an enhanced and dysregulated reparative process featuring emergence and alveolar migration of oxidative stress-resistant club cells.

### Atp8b1^G308V/G308V^ mice exposed to hyperoxia develop postinflammatory pulmonary fibrosis during the recovery phase

**Figure 5:**
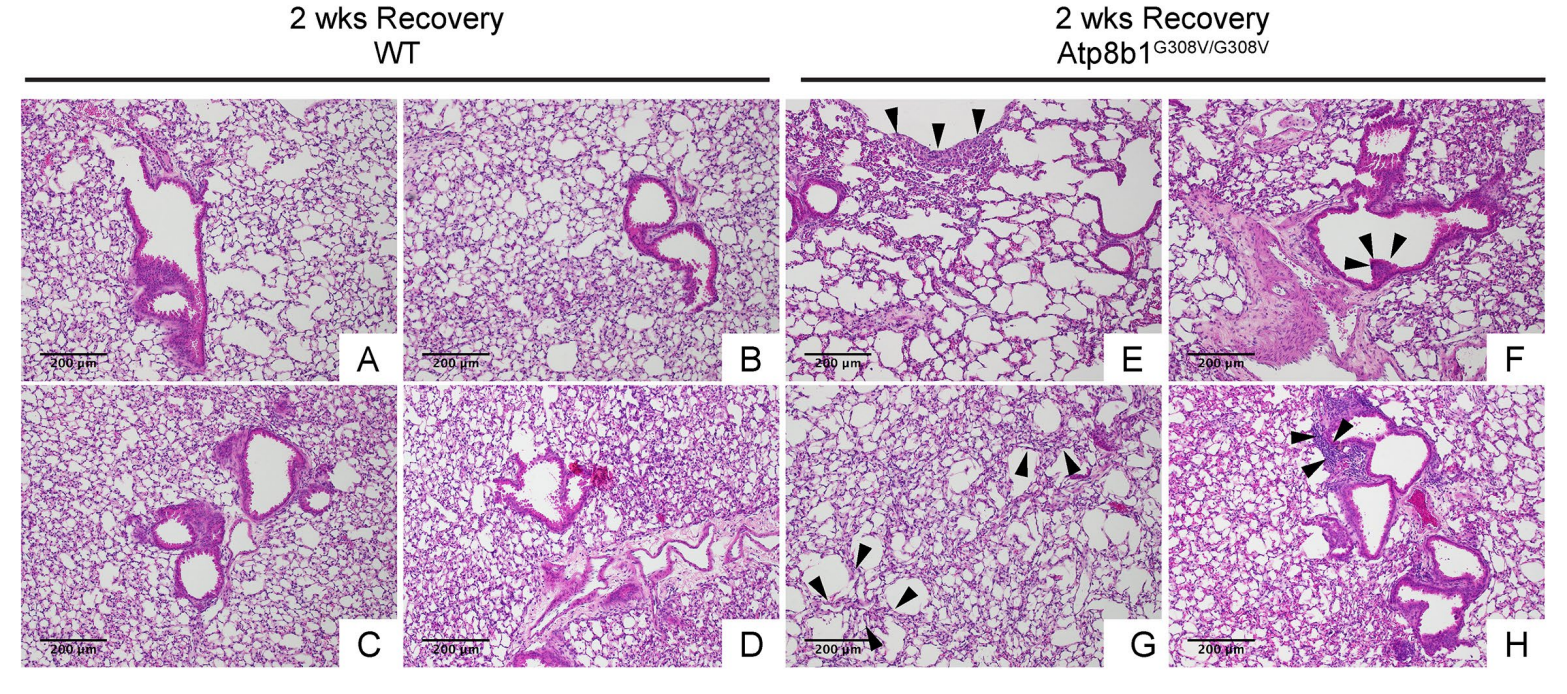
**Atp8b1^G308V/G308V^ mice exposed to hyperoxia and returned subsequently to normoxia for recovery develops late-onset interstitial fibrosis.** Representative photomicrographs of H&E-stained lung sections from 7-9-wk-old WT (**A-D**) and Atp8b1^G308V/G308V^ mice (**E-H**) that were exposed to 100% O_2_ for 48 hrs and allowed to recover under normoxia for 12 days (n=3 for each). WT mice show marked recovery with some hypercellularity remaining in bronchiolar regions. Atp8b1^G308V/G308V^ lungs display juxtaposition of normal lung with collapsed alveoli beneath the pleura (arrows in Panel E), distinct hyperplastic epithelium (arrowheads in F), thick-walled cystic air space (arrowheads in Panel G), and hypercellularity in bronchovascular interstitium (arrowheads in Panel H). Magnifications: (A-H) 100X. Data presented are representative of two independent experiments.

**Figure 6:**
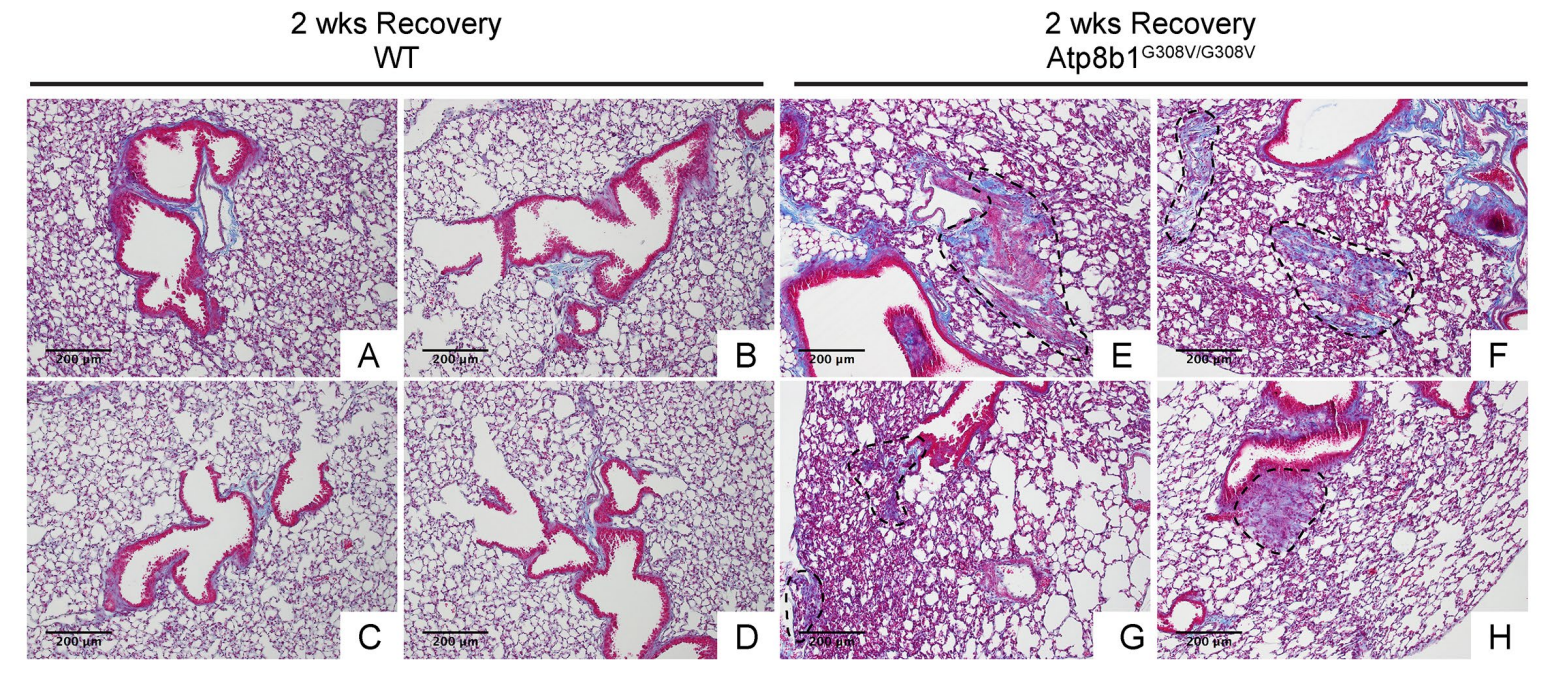
**Atp8b1^G308V/G308V^ mice exposed to hyperoxia and returned subsequently to normoxia for recovery display abnormal fibrotic reactions in the lung.** Representative photomicrographs of Masson's Trichrome-stained lung sections from 7-9-wk-old WT (**A-D**) and Atp8b1^G308V/G308V^ mice (**E-H**) that were exposed to 100% O_2_ for 48 hrs and allowed to recover under normoxia for 12 days (n=3 for each). WT mice show minimal collagen deposition mainly at peribronchiolar and perivascular areas. Atp8b1^G308V/G308V^ mice show patchy distribution of aberrant collagen deposition (areas circled by dashed lines in Panel E, F, G & H): (Panel E) perivascular region, (Panel F) alveoli, (Panel G) alveoli located at subpleura and alveoli adjacent to alveolar duct, and (Panel H) peribronchiolar region. Magnification: (A-H) 100X. Data presented are representative of two independent experiments.

**Figure 7:**
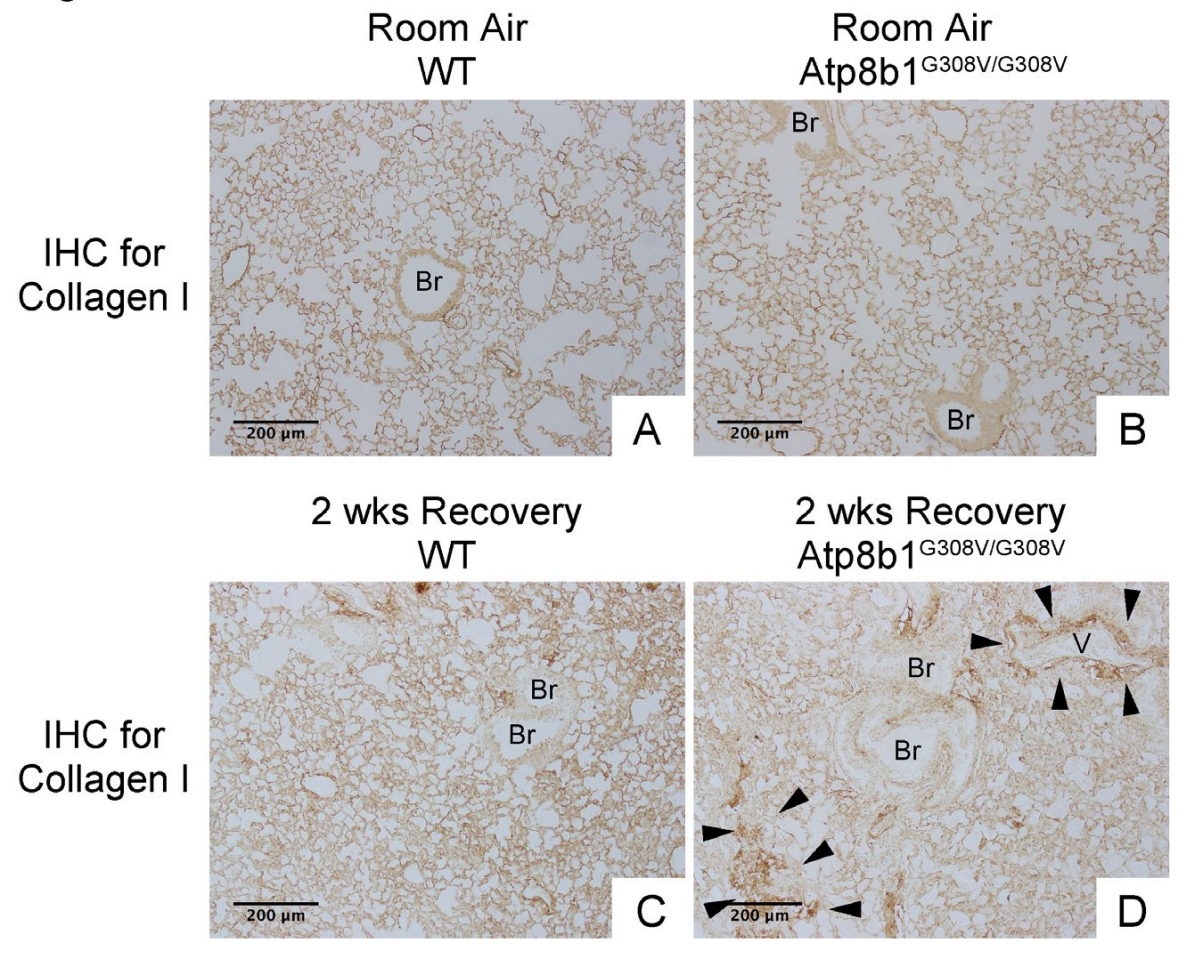
**Atp8b1^G308V/G308V^ mice exposed to hyperoxia and returned subsequently to normoxia for recovery display aberrant deposition of collagen in the lung.** Photomicrographs of lung sections immunohistochemically labeled for type I collagen. WT and Atp8b1^G308V/G308V^ mice at 7-9 weeks of age were exposed to room air or 100% O_2_ for 48 hours, and then allowed to recover under normoxia for 12 days (n=3 for each). Arrowheads indicate areas showing strong signals for type I collagen. Atp8b1^G308V/G308V^ mice display aberrant collagen deposition in both perivascular and alveolar regions. Br: Bronchiolar lumen; V: Vessel (bronchiolar artery). Magnification: (A-D) 100X. Data presented are representative of two independent experiments.

In IPF lungs epithelial cell apoptosis and hyperplasia are often encountered in close proximity to fibrotic lesions. Based on our findings that Atp8b1^G308V/G308V^ mice exhibit accelerated cell apoptosis in alveoli and enhanced proliferation of club cells under oxidative conditions, we speculated that Atp8b1^G308V/G308V^ mice exposed to oxidative stress would display late-onset fibrotic reactions which have a certain pathological similarity to IPF. To test this speculation, WT and Atp8b1^G308V/G308V^ mice were exposed to 100% O_2_ for 48 hrs and then allowed to recover under normoxia for 12 days. At day 14, lung tissues were collected for evaluating recovery from hyperoxic exposure. Assessment of the morphological phenotype using H&E-stained lung tissue sections revealed that Atp8b1^G308V/G308V^ mice at day 14 display non-uniform distortion of lung architecture. Strikingly, IPF-defining pathologic features were noted in Atp8b1^G308V/G308V^ lungs including (i) cystic lesions with thickened interstitium (arrowheads in Fig. 5G) and (ii) juxtaposition of normal lung with fibrotic lesions (arrowheads in Fig. 5E). Patchy proliferation of bronchiolar epithelium observed in Atp8b1^G308V/G308V^ mice at 48 hrs of hyperoxia still remained at day 14 even though its density was very low compared to 48 hrs (arrowheads in Fig. 5F). Meanwhile, WT mice on day 14 recovery phase, exhibited mostly normal lung structure compared to Atp8b1^G308V/G308V^ mice (Fig. 5A-D). To determine whether loss of normal lung architecture observed in Atp8b1^G308V/G308V^ mice exposed to hyperoxia and subsequent normoxia involves collagen deposition, lung tissues were stained with Masson-Trichrome method as well as immunohistochemical labeling for type I collagen. The results show that Atp8b1^G308V/G308V^ mice at day 14 display abnormal collagen deposition in perivascular, peribronchiolar, and alveolar regions (Fig. 6A-H; Fig. 7A-D). Collectively, these results suggest that hyperoxic insult-induced oxidative stress followed by normoxic recovery period causes Atp8b1^G308V/G308V^ lung to recapitulate a certain pathological process shared by IPF.

### Hyperoxia-induced late-onset pulmonary fibrosis developed in Atp8b1^G308V/G308V^ mice features impaired regeneration of normal bronchioalveolar structures

**Figure 8:**
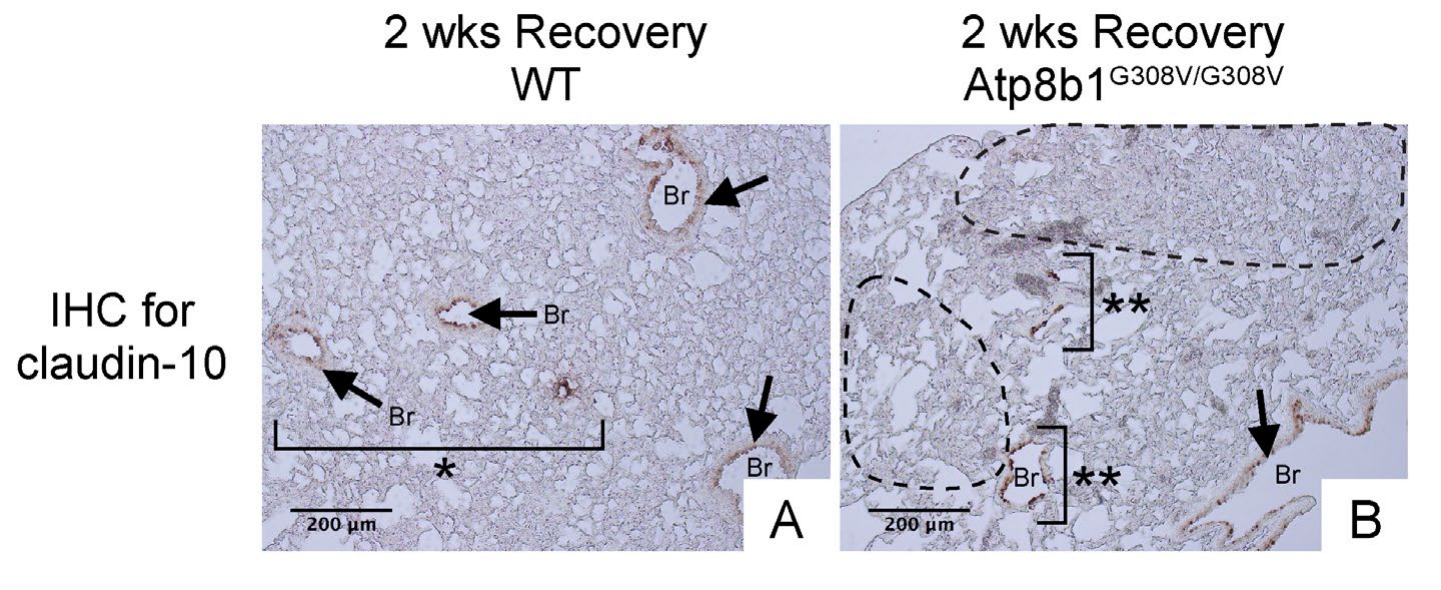
**Atp8b1^G308V/G308V^ mice exposed to hyperoxia and returned subsequently to normoxia for recovery show impaired bronchiolar regeneration.** WT and Atp8b1^G308V/G308V^ mice at 7-9 weeks of age were exposed to room air or 100% O_2_ for 48 hours, and then allowed to recover under normoxia for 12 days (n=3 for each). Representative photomicrographs of lung sections immunohistochemically labeled for claudin-10 are shown. Arrows denote relatively intact bronchiolar lumens. (**A**) WT lungs recovered from hyperoxia display normal regeneration of bronchioalveolar structures wherein organized arrangements of bronchioles surrounded by intact alveoli are noted (area designated by one asterisk). (**B**) Atp8b1^G308V/G308V^ lungs recovered from hyperoxia display impaired regeneration of bronchioalveolar structures wherein incomplete bronchiolar structures (two asterisks) are juxtaposed to highly remodeled lesions (areas circled by dashed lines). Br: Bronchiolar lumen. Magnifications: (A and B) 200X. Data presented are representative of two independent experiments.

Since Atp8b1^G308V/G308V^ mice develop patchy proliferation of oxidative stress-resistant club cells at early phase under hyperoxia followed by fibrotic reactions at late phase under normoxia, we hypothesized that in Atp8b1^G308V/G308V^ lungs these migratory club cells remain in alveoli and trigger aberrant fibrotic reactions. To elucidate the potential involvement of migratory club cells in oxidative stress-induced pulmonary fibrosis, WT and Atp8b1^G308V/G308V^ lungs at day 14 recovery phase were immunohistochemically labeled for claudin-10. The results show that well-organized bronchioles or bronchioles in relatively unaffected areas from both WT and Atp8b1^G308V/G308V^ lungs, exhibit low levels of claudin-10 (arrows in Fig. 8A & B). Severely affected areas with no discernible bronchiolar structures, which were often encountered in Atp8b1^G308V/G308V^ lungs, displayed no claudin-10 positive cells (areas circled by dashed line in Fig. 8B). Remarkably, however, cell clusters with strong claudin-10 signals forming incomplete bronchiolar structures were noted on the edges of severely affected lesions (double asterisks in Fig. 8B). These results suggest that dysregulated behavior of claudin-10-positive club cell clusters is the leading edge of fibrotic lesions with impaired reconstruction of bronchioalveolar structures seen in Atp8b1^G308V/G308V^ lungs.

### IPF lungs show aberrant CCSP expression at bronchiolar epithelium with hypercellularity

**Figure 9:**
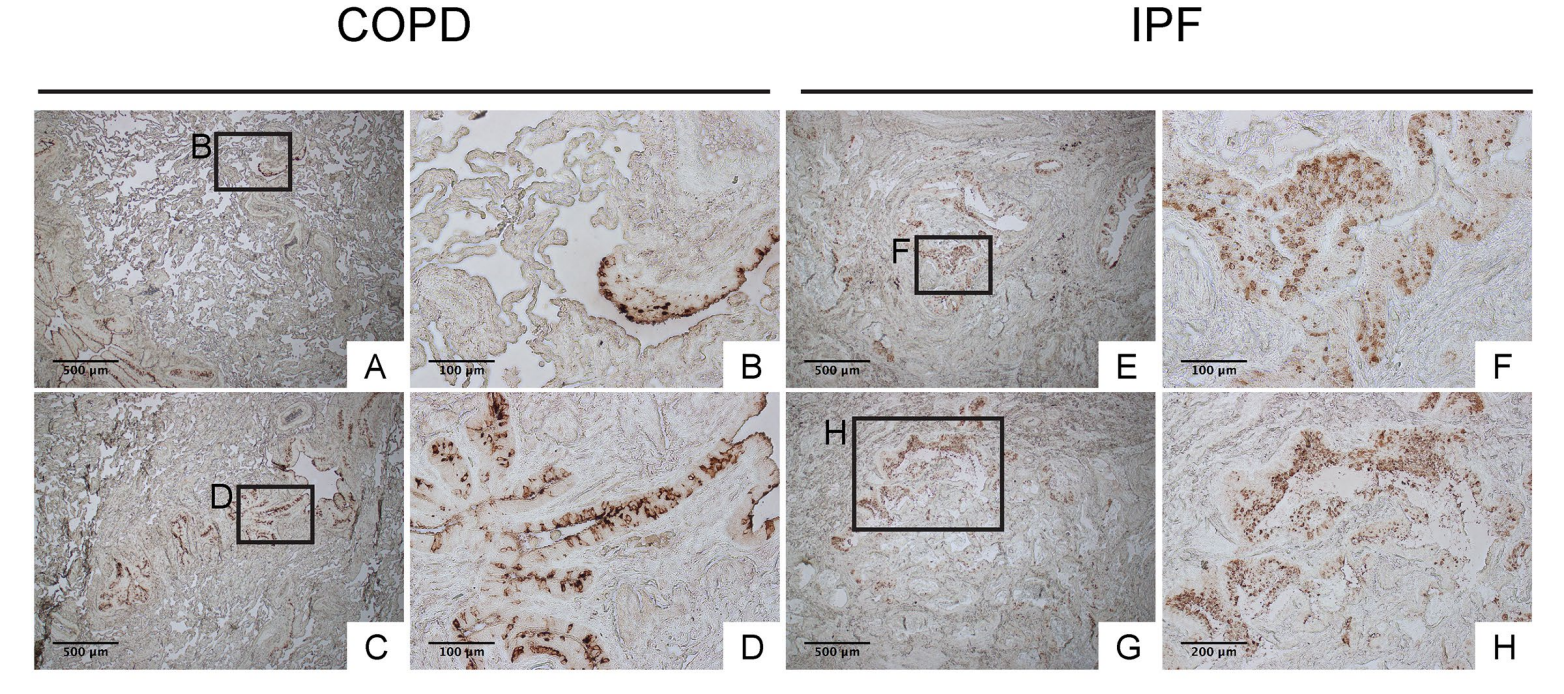
**IPF lungs show aberrant CCSP expression at bronchiolar epithelium with hypercellularity.** Immunohistochemical staining for CCSP was performed on lung tissue sections from patients with IPF and patients with COPD (control for IPF) (n=4 for each). Labeled boxes correspond to their respective enlarged images. (**A, B, C** & **D**) Representative photomicrographs of lung sections from COPD patients display relatively organized arrangement of CCSP expression in apical side of bronchiolar epithelium. (**E, F, G** & **H**) Representative photomicrographs of lung sections from IPF patients. Hyperplastic bronchiolar epithelium randomly displays accumulation of cells with varying degrees of CCSP expression. Magnifications: (A, C, E, G) 40X; (H) 100x; (B, D, F) 200X. Data presented are representative of one experiments.

**Figure 10:**
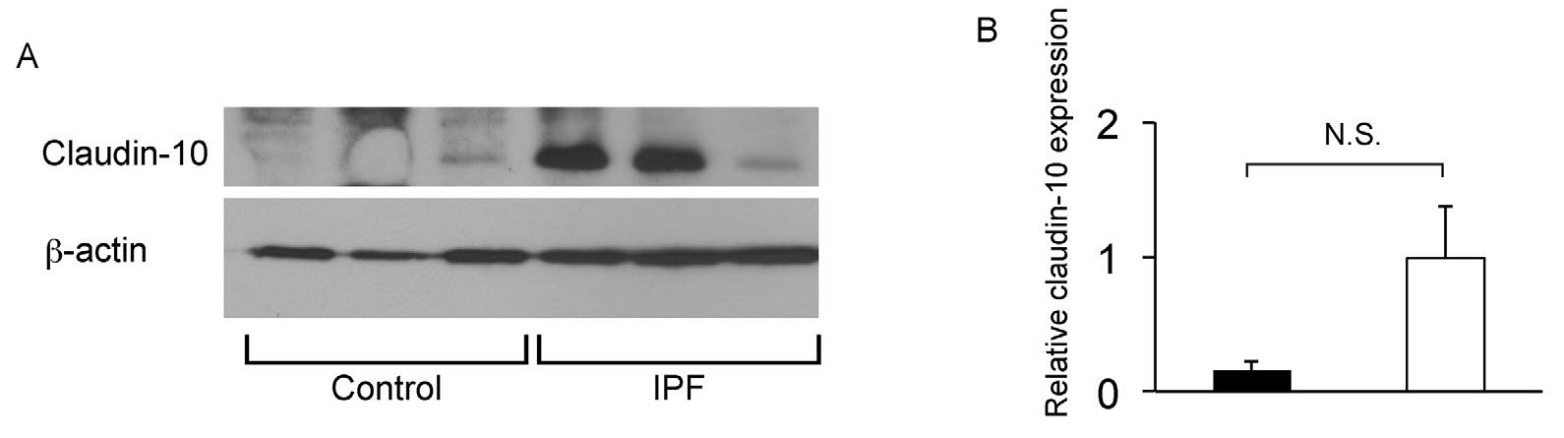
**IPF lungs show increased claudin-10 expression.** (**A**) Western Blot analysis was performed using whole protein lysates from IPF and control lung samples (n=3 for each), to determine relative abundance of claudin-10. Equal amounts of protein (50μg) were loaded per lane. Data presented are representative of two independent experiments. (**B**) Expression of claudin-10 in A was normalized to β-actin and presented in arbitrary units.

To confirm that club cell abnormality is associated with aberrant lung fibrosis, immunohistochemical labeling for club cell secretory protein (CCSP) was performed with focus on bronchiolar epithelium with hypercellularity. Since tissue sections from healthy lung could not be procured, those from COPD patients were used as controls. In COPD lung, which was used as a control group for IPF, CCSP signals were mainly detected in the bronchiolar epithelium in an organized manner (Fig. 9B & D). In IPF lung, however, its robust expression was found in a disorganized manner particularly in hyperplastic bronchiolar epithelium (Fig. 9F & H). These results are compatible with our previous work showing abnormal distribution and arrangement of claudin-10-positive club cells in IPF lung [6]. To corroborate the relationship of the abnormal behavior of club cells with IPF pathogenesis, the amounts of claudin-10 were examined using western blotting. The results show that claudin-10 expression is remarkably high in two out of the three IPF lung samples tested compared to healthy control lungs (Fig. 10). Taken together, it is suggested that alteration of the epithelial cell properties of club cells and/or other bronchiolar cell types, is closely linked to the pathogenesis of IPF.

### Lung epithelial cells with common subcellular ultrastructures are found in bronchiolar and alveolar epithelium in IPF lung

The similarities between hyperoxia-induced lung fibrosis in Atp8b1 mutant mice and IPF suggest that damage-induced migratory bronchiolar epithelial cells proactively give rise to metaplastic epithelial cells and thereby prompt fibrosis in alveoli. We hypothesized that there are similarities regarding the ultrastructural morphology between alveolar epithelial cells and metaplastic bronchiolar epithelial cells in IPF lungs. To test this hypothesis, we characterized the structure of various epithelial cells in IPF lungs using transmission electron microscope (TEM).

**Figure 11:**
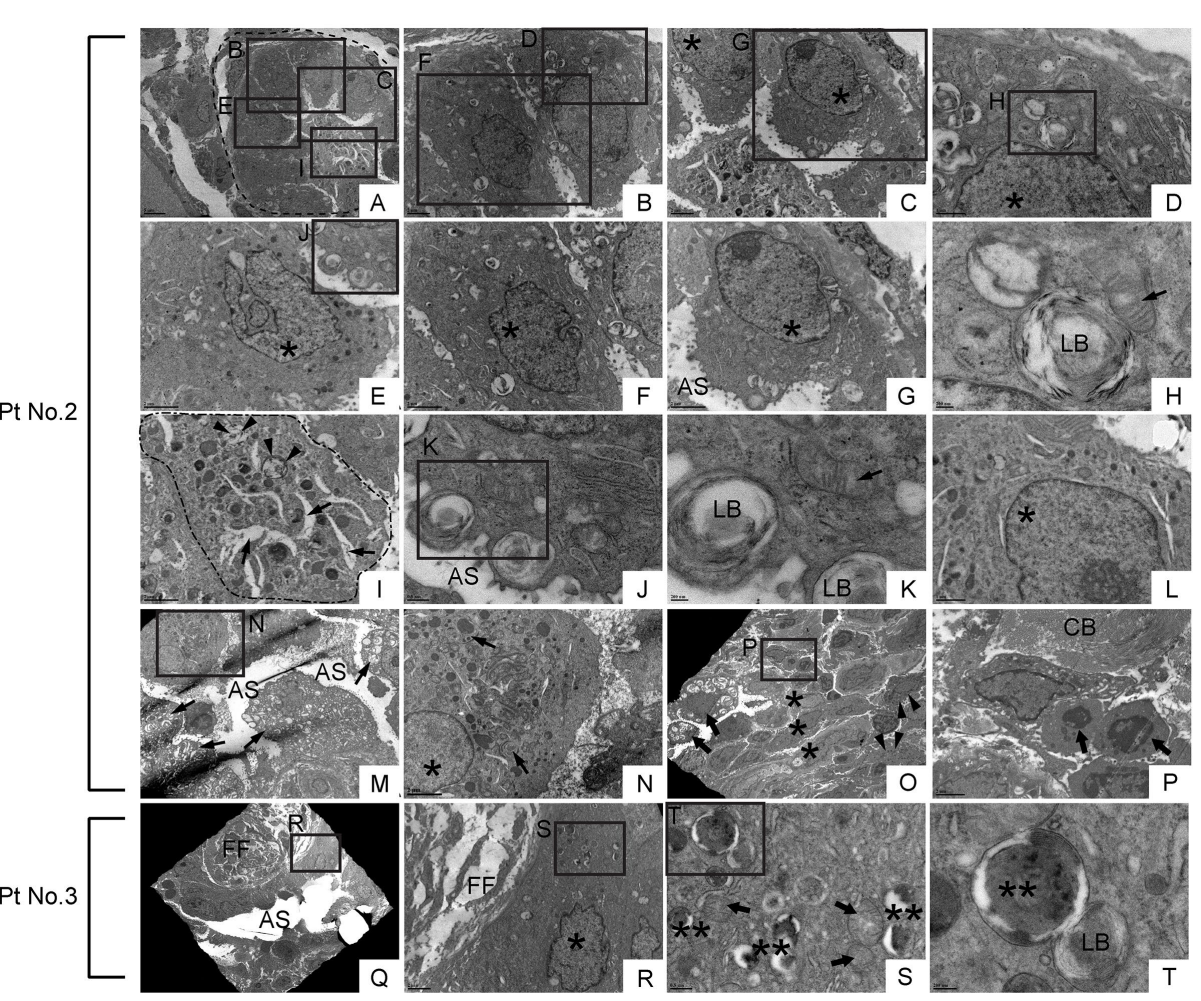
**Dysmorphic lamellar bodies, cytoplasmic vacuolation, and euchromatic nucleus are widely shared in metaplastic bronchiolar epithelial cells in IPF lung.** Transmission electron microscopy was performed on lung samples from patients with IPF (n=5). Data presented are from Patient No. 2 and Patient No. 3. Labeled boxes correspond to their respective enlarged images. (**A** & **I**) A small bronchiole (circled by dashed line in Panel A) is occluded by a suspected cell structure featuring multiple granules, lamellar bodies (arrowheads in Panel I), and an enlarged endoplasmic reticulum (arrows in Panel I). (**B-G** & **L**) Bronchiolar epithelial cells exhibit morphological variation with frequently encountered features of euchromatic nucleus (asterisks in Panel C, D, E, F & G) and dysmorphic lamellar bodies. (**H**) A dysmorphic lamellar body next to a trace of mitochondria. (**J** & **K**) Lamellar bodies present at the apical surface of a bronchiolar epithelial cell are about to secrete their contents into the airspace. An arrow in Panel K denotes mitochondria. (**M**) A narrowed bronchiolar lumen lined by highly vacuolated epithelial cells with dysmorphic lamellar structures (arrows). (**N**) A cell with euchromatic nucleus (asterisk) and morphological feature of club cell (numerous secretory granules in cytoplasm) is located in bronchiolar epithelium. Prominent phagosomes are noted in cytoplasm (arrows). (**O** & **P**) Hyperplastic bronchiolar epithelium with collagen deposition in the interstitium. Cells with euchromatic nucleus (asterisks in Panel O) are located across apoptotic cells (arrows in Panel P) from collagen bundle bundles (CB). Cells with numerous secretory granules in cytoplasm, which are considered to be club cells, are noted (arrowhead in Panel O). Seen at luminal side are cells with numerous lamellar structures in cytoplasm (arrows in Panel O). (**Q-T**) Metaplastic epithelial cell on fibroblastic foci features dysmorphic mitochondria (arrows in Panel S), numerous phagosomes (double asterisks in Panel S & T), and a small number of lamellar bodies. AS: Airspace; FF: Fibroblastic foci; LB: Lamellar body. *(single asterisk) = Euchromatic nucleus, **(double asterisks) = Phagosome. Magnifications: (A) 3000X; (B, C, and R) 8000X; (D & L) 20000X; (E, F, G, I, N & P) 12000X; (H & K) 60000X; (J & S) 30000X; (M) 4000X; (O) 2500X; (Q) 1500X; (T) 80000X.

In relatively small bronchioles in IPF lung, which anatomically are categorized as terminal or respiratory bronchioles, we encountered cells with club cell morphology [i.e. electron-dense granules, or secretory granules, in cytoplasm] (Fig. 11E, I, L & N). However, they displayed huge disparities in size as well as the number of secretory granules. Additionally, their epithelial polarity was lost and they displayed a small number of suspected lamellar bodies in their cytoplasm (arrowheads in Fig. 11I). Adjacent to atypical club cells in terminal or respiratory bronchioles with atypia, were euchromatic cells with traces of dysmorphic lamellar bodies in small numbers (Fig. 11B, C, D, F, G, H & J) or highly vacuolated epithelial cells with dysmorphic lamellar bodies in relatively large numbers (arrows in Fig. 11M). These results suggest that in terminal or respiratory bronchioles in IPF lungs, club cells, and certain lineage-committed progenitors, are actively differentiating towards type II AECs. It is unclear whether the euchromatic cells spotted adjacent to atypical club cells are giving rise to club cells or the other way around. In relatively large and/or thickened metaplastic bronchioles, cells with club cell morphology were rarely found (arrowhead in Fig. 11O). These TEM observations, that cells with club cell morphology are mainly spotted in small bronchioles of IPF lungs, is consistent with the normal histology of human lungs wherein club cells mainly reside at terminal to respiratory bronchioles. In relatively large and/or thickened metaplastic bronchioles, two distinct cell types and morphologically intermediate cells were observed occupying the large portion of the epithelium. One type is morphologically simple cells showing a nucleus with extremely clear chromatin, high nucleus-to-cytoplasm (N/C) ratio, and minimal vacuolation in the cytoplasm (asterisk in Fig. 11O; cells in an area circled by a dashed line in Supp. Fig. 1H; asterisks in Supp. Fig. 1L). Another type of predominant cells possesses a nucleus showing varying degrees of chromatin condensation, highly vacuolated cytoplasm, and dysmorphic lamellar bodies (arrows in Fig. 11O & P; arrows in Supp. Fig. 1E, I & L). Noticeably, the epithelial cells covering the fibroblastic foci were largely “intermediate cells” (Fig. 11Q-T). Quite intriguingly, some of the above mentioned vacuolated cells were spotted in the airspace, suggesting that they may be actively recruited to damaged alveoli through airspace (arrow in Fig. 11O; arrowheads in Supp. Fig. 1F, G & K; arrows in areas circled by dashed lines in Supp. Fig. 1E & I). These findings suggest that in IPF lungs, lung-specific progenitor cells committed to type II AECs are actively proliferating and recruited to damaged alveoli.

**Figure 12:**
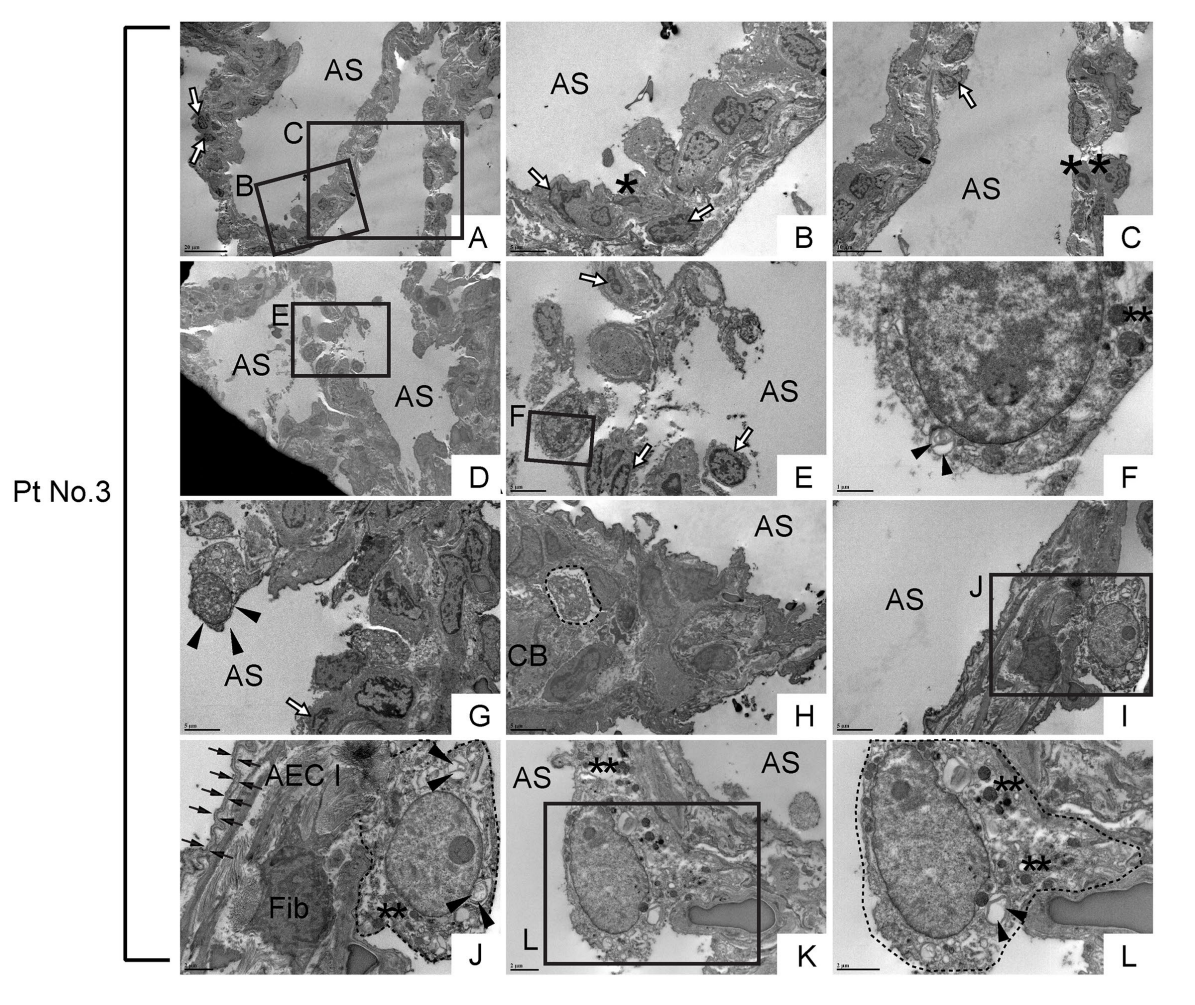
**Morphologically abnormal type II alveolar epithelial cells with similar subcellular structures to metaplastic bronchiolar epithelial cells are encountered in alveoli of IPF lung.** TEM was performed on human IPF samples from patient No. 3. Labeled boxes correspond to their respective enlarged images. (**A-E**) Alveolar regions with slight interstitial fibrosis show random distribution of cells with varying degrees of apoptosis. Cells with highly condensed chromatin, which are considered to be at late stages apoptosis, are designated by asterisks while cells exhibiting narrow cytoplasm and marginal condensation of chromatin, which are considered to be at early stages of apoptosis, are designated by open arrows. (**F**) Floating in airspace is a highly atypical cell featuring high nucleus-to-cytoplasm (N/C) ratio, dysmorphic lamellar body (arrowheads) and granular structures suspected of either secretory granules or degraded mitochondria (double asterisks). Note the appearance of cytoplasm and cytoplasmic membrane reminds loss of cell viability meanwhile the nucleus does not show the features of apoptosis. (**G**) A highly atypical cell with a lot of granular structures is barely attached to the alveolar tissue as if it has migrated and just landed on alveolar tissue. (**H**) A suspected epithelial cell type (circled by dashed line) featuring microvilli and dysmorphic lamellar bodies, quite similar to the highly vacuolated bronchiolar epithelial cells seen in bronchiolar region (arrows in Fig. 11M). (**I-L**) Metaplastic epithelial cells suspected of either type II AECs or progenitor cells committed to type II AECs (circled by dotted line) featuring dysmorphic lamellar bodies (arrowheads), granular structures, slightly euchromatic nucleus and vacuolated cytoplasm. Note the juxtaposition of collagen producing active fibroblast (Fib) with a metaplastic epithelial cell with type II AEC morphology (Panel J), which suggests epithelial-to-mesenchymal interaction. Type I AEC (arrows in Panel J) across the active fibroblast from metaplastic epithelial cell show relatively intact appearance. AS: Airspace; AEC I: type I alveolar epithelial cell; CB: Collagen Bundle; Fib: Fibroblast. *(single asterisk) = Apoptotic cell, **(double asterisks) = Granular structures suspected of secretory granules or degraded mitochondria. Magnifications: (A and D) 1200X; (B, E, G, H & I) 4000X; (C) 2500X; (F) 20000X; (J and K) 8000X; (L) 12000X.

In alveolar areas with mild fibrosis in IPF lung, a lot more cells with varying degrees of apoptosis were noted in random distribution (asterisks and open arrows in Fig. 12A, B, C, E & G), which is in contrast to the hyperplastic bronchiolar epithelium where the apoptotic cells are rarely encountered. Cells with typical club cell morphology were not found in alveoli. Instead, cells with vacuolation and dysmorphic lamellar bodies were often encountered (Fig. 12F; cells in areas circled by dashed lines in Fig. 12H, J & L), which exhibit morphological similarities to epithelial cells found in metaplastic bronchiolar epithelium. However, types II AEC-looking abnormal epithelial cells found in IPF alveoli often have nucleus with relatively condensed chromatin compared to abnormal epithelial cells in bronchiolar epithelium. Many of these morphologically abnormal alveolar epithelial cells are either barely attached or half-attached to the alveolar architectures (arrowhead in Fig. 12G; cells circled by dashed lines in Fig. 12H, J & L) implying that these cells have been recruited from bronchiolar epithelium to the damaged alveoli in IPF lung. Intriguingly, morphologically abnormal “half-attached epithelial cells” occasionally showed juxtaposition to apparently active fibroblasts surrounded by abundant collagen fibers (Fig. 12J). Taken together, these TEM observations of IPF lungs support our prior assumption that in IPF lungs damage-induced migratory bronchiolar epithelial cells proactively give rise to metaplastic alveolar epithelial cells and thereby prompt fibrosis in intact alveoli.

## DISCUSSION

In the current study, we have shown for the first time that Atp8b1^G308V/G308V^ mice, when exposed to oxidative stress (hyperoxia), exhibit accelerated club cell proliferation at bronchiolar epithelium and enhanced cell apoptosis at alveolar epithelium. These ambivalent hyperoxia-induced responses that occurred in Atp8b1 deficient lungs were followed at the recovery phase by non-uniform fibrosis, affecting both the peribronchiolar and alveolar areas. We further bridged the Atp8b1^G308V/G308V^ mice-based hyperoxia fibrosis model and human IPF pathology by revealing that IPF lungs display aberrant arrangement of CCSP-positive club cells in hyperplastic bronchiolar epithelium as well as increased claudin-10 expression in the lung. Consistently, TEM-assisted characterization of subcellular structures of metaplastic lung epithelial cells provided evidence supporting the notion that migratory bronchiolar epithelial cells are potential key players in IPF pathogenesis.

A recent paradigm shift labeled IPF as a multifactorial disease; a genetic predisposition to abnormal AEC regulation combined with other environmental and/or aging related factors [19–21]. Based on this conceptual shift, concerted efforts have been made to generate a “two-hit” IPF model that is both feasible and reproducible. We have demonstrated here that a specific Atp8b1 gene mutation, coupled with hyperoxic exposure in a murine model, recapitulates a series of pathological events that commences with apoptosis and proliferation of lung epithelial cells and leads to pulmonary fibrosis. However, fibrotic changes that develop in hyperoxia-exposed Atp8b1^G308V/G308V^ mice mainly affect the peribronchiolar regions; a finding not pathognomonic for IPF. Our fibrosis model does not exhibit some of the key pathological features of IPF: i.e. (i) cystic change more prominent at the periphery and base of the lung, (ii) existence of fibroblastic foci, morphologically distinct foci of active fibroblasts juxtaposed with metaplastic epithelial cells, occurring at the interface between dense scar and adjacent normal lung, (iii) patchy subpleural fibrosis at early stages in the disease process, and (iv) extensive perilobular fibrosis with scaring of the centrilobular areas. A plausible reason for this pathological discrepancy between our model and human IPF is that mouse lungs anatomically correspond to the lower part of human lungs [22], and hyperoxia-induced oxidative stress affects the whole mouse lung evenly as opposed to environmental insults to human lungs that can be more intense in distal than proximal regions [23,24].

Atp8b1 is a membrane-bound transporter for cardiolipin in type II AECs that localizes to the apical membrane of epithelial cells [25–27]. G308V mutation causes functional deficiency in Atp8b1 through aberrant folding of protein in endoplasmic reticulum and decreased expression of the protein in plasma membrane [12,28]. Therefore, G308V mutant Atp8b1 proteins expressed in type II AECs are functionally deficient as cardiolipin transporters. However, it remains unclear how Atp8b1 deficiency provokes cell type-dependent ambivalent responses, i.e. proliferation and apoptosis under hyperoxic conditions. The fate of each lung epithelial cell type under hyperoxic conditions may be affected by its differentiation status and/or apoptosis threshold. Our preliminary studies using immunohistochemical labeling on lung tissue sections show that Atp8b1 is expressed in bronchiolar as well as alveolar epithelium (data not shown). Since cardiolipin is a major component of the mitochondrial inner membrane and maintenance of cardiolipin homeostasis is a key determinant of mitochondrial integrity [13,29,30], we speculated that alveolar and bronchiolar epithelial cells would be equally affected by G308V mutation in Atp8b1. The observed results show that this speculation is not correct. It seems conceivable that functionally differentiated type II AECs undergo apoptosis under hyperoxic conditions meanwhile club cells survive oxidative conditions and give rise to type II AECs. Loss-of-function mutation in Atp8b1 may accelerate this response and provoke persistent fibrotic responses. At this juncture, we have not analyzed cardiolipin levels in lungs or BAL fluid in our fibrosis model. Future studies focusing on the interplay between the airway cardiolipin levels and fibrotic responses would inform whether elevated airway cardiolipin is a critical regulator in the fibrotic responses that develop in hyperoxia-exposed Atp8b1^G308V/G308V^ mice.

Club cells, also known as “nonciliated secretory cells”, are a multifunctional cell type. The main roles for club cells are i) regeneration [31–34], ii) immunomodulation [35] and iii) detoxification [36]. Varied attempts in pursuit of the mechanism of lung regeneration have highlighted club cells as an important progenitor cell type that is actively involved in the complex and harmonized regeneration process of the lung: e.g. i) club cells can self-renew and give rise to ciliated, goblet and type II alveolar epithelial cells [10,31,37–39], ii) club cells implement their regenerative functions in close cooperation with other progenitor cells [34,40–42] and iii) there exist subsets of CCSP-positive cells that are labeled for other lineage marker(s) such as p63 and prosurfactant protein C (pro-SPC) [43,44]. The quantitative profile on hyperoxia-induced proliferation of club cells was first documented in 1978 using rat, where the authors revealed that club cells can proliferate under 80% O_2_ [32]. Whether club cells can proliferate under even higher fractions of inspired oxygen has not been examined so far. Instead, injurious effects of hyperoxia, e.g. cellular and humoral inflammation as well as epithelial cell death, have been focused and emphasized [45–47]. Accordingly, hyperoxic insult has not been considered as a tool for inducing club cell proliferation, not to mention a tool for inducing IPF-relevant pulmonary fibrosis. Indeed, neither the lungs from patients who die after extended exposure to hyperoxia, nor the animals exposed to long-term hyperoxia, show any histopathological similarities to IPF [48,49]. However, a previous report has demonstrated that markers for oxidative stress are spotted in metaplastic epithelial cells in IPF lung [50]. In addition, evidence has revealed that club cells, which can proliferate under highly oxidative conditions ([32]; Fig. 4A-F), display varied cell morphology and wide distribution in IPF lungs [6]. Collectively, the use of hyperoxia at a moderate intensity is a theoretically reasonable option to induce a potentially important pathological aspect of IPF, i.e. the dedifferentiation and proliferation of club cells [6].

The major oxygen toxicity to the living organisms is not caused directly by the O_2_ molecule, but rather through catalytic intermediates such as free radicals generated during the metabolic processing of O_2_ [51]. Oxidation of lipids, proteins, and nucleic acids caused by highly reactive radicals lead to a loss of integrity in membrane structure, deactivation of numerous enzymes, altered gene expressions, and, ultimately, cell death [51]. Therefore, it is the mitochondrial’s ability to produce energy source through the use of O_2_ that jeopardizes the survival of mitochondria-containing cells. The antioxidative response of each lung epithelial cell type during exposure to and recovery from hyperoxia is not well characterized. Meanwhile, the activity of the mitochondria-localized antioxidant enzyme manganese superoxide dismutase (Mn-SOD) in the whole lung is known to decrease during the initial 48 h of hyperoxia and increase during the subsequent 24 h of normoxic recovery [52]. This physiologically unreasonable decrease of Mn-SOD in the lung during hyperoxia is intriguingly compatible with the *in vitro* study, showing that cytokine-induced Mn-SOD does not protect bronchial epithelial cells against oxidants, but rather makes them vulnerable to oxidative damage [53]. In light of such background, we surmise that the proliferative club cells observed under hyperoxia in the current study are the cells which had survived hyperoxic conditions by suppressing mitochondrial energy production and the activity of Mn-SOD. This hypothesis is compatible with the TEM observation that in IPF lungs occasionally encountered non-apoptotic and non-necrotic lung epithelial cells display a paucity of mitochondrial cristae, a fold of the inner membrane that provides locations for ATP production (data not shown). The reason why Atp8b1^G308V/G308V^ mice exhibit an enhanced proliferation of club cells under hyperoxia is not clear. One possible explanation is that the reservoir of progenitor cells that are committed to become club cells expands spontaneously in a random and patchy manner in the lungs of Atp8b1^G308V/G308V^ mice. This could theoretically be due to an inherent response of club cells to replace spontaneously damaged type II AECs, which are functionally more differentiated and accordingly more vulnerable to Atp8b1 deficiency. Slightly thickened bronchiolar epithelium that is randomly seen in Atp8b1^G308V/G308V^ mice under normoxia (Fig. 7B) may reflect an expansion of such primed progenitor cells. Atp8b1^G308V/G308V^ mice exhibited impaired bronchioalveolar structures and associated fibrotic reactions at the recovery phase after hyperoxia in our study. The most plausible scenario for this observation is that, due to the lack of functional Atp8b1, restoration of normal bronchioalveolar structures is impaired and lung fibrosis perpetuates in Atp8b1^G308V/G308V^ mice. We have no clues as to whether cardiolipin clearance ability of Atp8b1 plays a key role in lung regeneration after hyperoxia. There may be unrevealed roles for Atp8b1 that is related to mitochondrial damage and their recovery.

Oxidative stress and redox imbalance have been among the therapeutic targets for the treatment of IPF [54–56]. In our animal model, oxidative stress, i.e. hyperoxia, is a key factor that induces alveolar epithelial damage and club cell proliferation. Genetic predisposition, i.e. Atp8b1 mutation is another key factor that is suspected to contribute to the priming of club cells and delayed regeneration of alveoli. Due to such characteristic profiles, our animal model holds a potential to be used as a versatile platform in the research of IPF. As an example, a newly developed antifibrotic drug can be tested in our model: the drug could be administered day 7 through day 14 after hyperoxia to see if it suppresses ongoing fibrosis, and day 14 through day 21 to see if it alleviates established fibrosis. This platform also has the potential to reveal how lung progenitor populations, including club cells, quickly behave in response to each situation, i.e. in the midst of hyperoxia (24-48h), immediately after hyperoxia (48-72h), and at the recovery phase (day 7-14).

Dysmorphic lamellar structures are frequently encountered in metaplastic bronchiolar epithelial cells (Fig. 11 B, D & H; arrowheads in Fig. 11I; Fig. 11 J & K; arrows in Fig. 11M & O) and alveolar cells (arrowhead in Fig. 12F, J & L) in IPF lungs. Interestingly, those cells with dysmorphic lamellar structures often possess clear chromatin (asterisks in Fig. 11D & G; Fig. 12J & L), large nucleoli (Fig. 11G; Fig. 12J), and morphologically abnormal mitochondria (arrow in Fig. 11K; double asterisks in 12J & L). These observations suggest that i) the appearance of lung epithelial cells with abnormal lamellar bodies and mitochondria is a critical event in IPF pathology and ii) such cells with abnormal lamellar bodies and mitochondria have aberrant nucleolar activity induced by activation of oncogenes or persistent replicative stress [57]. Further investigations are required to clarify how cellular senescence affects lamellar body maturation and morphology of lung epithelial cells. Also, it would be interesting to determine if Atp8b1 deletion, not deficiency, is sufficient to elicit detrimental effects on lamellar body maturation and mitochondrial energy metabolism. This might be a solution to unravel the missing link between the mitochondrial dysfunction [58] and surfactant abnormality [59] observed in IPF.

One of the biggest challenges inherent to the TEM-assisted study is the stringent limitation of sample size and number that can be examined during a fixed period of time. To alleviate this limitation, we closely examined Toluidine Blue-stained lung sections that were cut from the resin-embedded lung samples used for TEM. In each of the 5 IPF lung samples tested, we found more than one bronchiolar area that was in close proximity to fibrotic lesions with abundant collagen bundles. In advanced fibrotic areas with dense collagen deposition, the lining epithelial cells are necrotic or barely detectable leaving almost denuded epithelium (data not shown), which is consistent with previous report [60]. In moderately fibrotic areas including fibroblastic foci, the lining epithelium showed a huge diversity in appearance. Since IPF lung sections labeled for claudin-10 display widespread of claudin-10-positive cells: i) accumulating in varied arrangement at hyperplastic bronchiolar epithelium and ii) floating as clusters in bronchiolar airspace [6], we had expected to find many cells with club cell morphology that are discernible under TEM observation. However, cells with club cell morphology were focally found in small metaplastic bronchioles, but neither in alveoli or airspace under TEM. This discrepancy between IHC and TEM findings may be reconciled by the explanation that club cells under severe environmental stress shift their status closer to type II AECs whereby they lose their club cell morphology, i.e. numerous cytoplasmic granules. This assumption is compatible with the observation that claudin-10 expression is detected throughout the developing lung epithelium and, as lung matures, converges to club cells [61].

Our TEM observations revealed that the distinct subcellular structures (cytoplasmic vacuolation and dysmorphic lamellar bodies) are commonly shared among metaplastic bronchiolar epithelial cells in fibrotic lesions and atypical type II AECs in relatively unaffected alveoli. Vacuolated atypical cells with or without dysmorphic lamellar bodies are found floating in both bronchiolar and alveolar airspace, as single cells (arrows in Fig. 11O, arrowheads in Supp. Fig. 1F, G & K) or as cell clusters (area circled by dashed line in Supp. Fig. 1E & I) in IPF lung. Intriguingly, morphologically similar vacuolated cells are spotted in airspace of hyperoxia-treated Atp8b1^G308V/G308V^ mice (Fig. 2E & F), which gives credit to our hyperoxia fibrosis model. We have no convincing conclusion what is the primary source site of these atypical cells seen in IPF lungs, either bronchiolar or alveolar epithelium. However, considering the observations that vacuolated cells (regardless of possession of lamellar bodies) are found with more frequency in metaplastic bronchioles and adjacent airspace, the former is the presumed major source site (Fig. 11E, F, G, J & M; Supp. Fig. 1E, H, I & L).

In summary, we present evidence for the first time that, in response to hyperoxic insult, Atp8b1^G308V/G308V^ mice show accelerated lung epithelial cell apoptosis in alveoli and augmented proliferation of oxidative stress-resistant club cells. This ambivalent response of Atp8b1^G308V/G308V^ lungs under oxidative conditions was followed at late recovery phase under normoxia by the development of aberrant fibrotic responses including impaired regeneration of bronchioalveolar structures. We also present here compelling data suggesting the clinical relevance of migrating bronchiolar epithelial cells by investigating ultrastructural similarities between metaplastic bronchiolar epithelial cells and atypical type II AECs in alveoli. Thus, the combination of genetic predisposition to epithelial dysfunction and hyperoxic insult will make a novel and promising platform upon which to study the role of bronchiolar epithelial migration in IPF. A more precise understanding of the cellular and molecular backgrounds of IPF pathophysiology utilizing this platform will unequivocally expedite the development of truly effective treatments against IPF.

## MATERIALS AND METHODS

### Mice

All procedures were approved by the Animal Care and Use Committee of University of South Florida. Atp8b1^G308V/G308V^ mice were a generous gift from L. Bull (University of California–San Francisco). C57BL/6J mice (Harlan laboratories, Indianapolis, IN) were used as controls. In all experiments, mice at 7-9 wks of age were used. During hyperoxic exposure, mice (n=4 for each group) were placed in cages in an airtight chamber (75×50×50 cm) and exposed to 100% O_2_. The oxygen concentration in the chamber was monitored and regulated with proOx P100 (BioSpherix, NY, USA). A portion of mice were euthanized 48 hrs after exposure to 100% O_2_ (acute inflammatory phase). The remaining mice were euthanized after an additional 12 days under normoxia (post-inflammatory recovery phase).

### Collection of mouse lungs

Mice were euthanized by intraperitoneal injection of a ketamine/xylazine mixture followed by cervical dislocation. Following thoracotomy, the inferior vena cava (IVC) was clamped and 2 ml of sterile phosphate-buffered saline (PBS) was injected into the right ventricle for lung perfusion. For histological analysis of the lung samples, the whole lung was inflation-fixed in 10% neutralized formalin at 20 cm H_2_O pressure. Formalin-fixed lungs were then embedded in paraffin. Thin sections were cut from the paraffin-embedded tissue blocks using microtome and stored at room temperature until use.

### Bronchoalveolar lavage analysis

Blood was removed from the IVC, as described above, followed by a small transverse incision on the skin of the ventral neck. The trachea was exposed, a catheter was inserted, and a whole-lung lavage was performed by using ice-cold sterile PBS (3 times, 1 ml each). Retrieved bronchoalveolar lavage (BAL) fluid, (2–2.5 ml), was centrifugated at 400g for 10 min at 4°C. Cell pellets were resuspended in 1 ml of ice-cold sterile PBS. Aliquots (200–400 μl) of cell suspension were centrifugated onto glass slides at 800 rpm for 3 min using a cytocentrifuge (Shandon Cytospin 2, Pittsburgh, PA). The cells on glass slides were stained using Diff-Quik stain set (Andwin Scientific, Schaumburg, IL).

### ELISA

The levels of interleukin-6 (IL-6) in BAL fluid were measured using the commercial ELISA kit (BD Biosciences, San Diego, CA) as per the manufacturer’s instructions.

### Human IPF and COPD samples

Human lung samples from patients with IPF and patients with COPD were obtained from the Lung Tissue Research Consortium (LTRC) funded by the National Institutes of Health (NIH). Human control lung samples were obtained from the National Disease Research Interchange (NDRI). Formalin-fixed and paraffin-embedded lung tissue sections from patients with IPF (n=4) and patients with COPD (n=4) were used for immunohistochemistry. Frozen lung samples from patients with IPF (n=3) and samples from healthy lungs (n=3) were used for Western blotting. Glutaldehyde-fixed lung samples from patients with IPF (n=5) were used for transmission electron microscopy (TEM).

### Routine and immunohistochemical staining on lung tissue sections

Routine morphological analysis was performed using lung tissue sections stained with hematoxylin and eosin (H&E) and the Masson-Trichrome (MT) method. Immunohistochemical (IHC) staining was performed on paraffin-embedded lung tissue sections. Briefly, paraffin sections were deparaffinized. Then, sections were subjected to heat-induced antigen retrieval (HIAR) in a 10 mM citric acid buffer (pH 6). Then, endogenous peroxidase activity was quenched with 3% hydrogen peroxide in PBS for 20 min and subsequently blocked with 10% goat serum for an additional 20 min. Sections were then incubated with either rabbit anti-club cell secretary protein (CCSP) antibody (EMD Millipore, Billerica, MA), rabbit anti-type I collagen antibody (Abcam, Cambridge, MA), or rabbit anti-claudin-10 antibody (Life Technologies, Carlsbad, CA). Next, the sections were incubated for 30 min with goat anti-rabbit IgG, which was conjugated with horseradish peroxidase (HRP) (Histofine Simple Stain Mouse MAX Peroxidase; Nichirei, Tokyo, Japan). Finally, detection of the target protein was performed using 3, 3’-diaminobenzidine (DAB) reagent.

### TUNEL Staining

TUNEL staining was performed on paraffin-embedded lung sections using an In Situ Cell Death Detection Kit, POD (Roche Applied Science, Indianapolis, IN), according to the manufacturer’s instructions with some modifications. Briefly, deparaffinized sections were permeabilized with 0.55 unit/ml proteinase K (Sigma-Aldrich, St. Louis, MO) for 45 min at 37 °C. After quenching with 3% hydrogen peroxide in PBS for 20 min, the sections were incubated for 60 min at 37 °C with a labeling mixture containing terminal deoxynucleotidyl transferase (TdT) and fluorescein-labeled nucleotides. After rinsing, the sections were incubated with sheep anti-fluorescein antibody conjugated with HRP for 30 min at 37 °C, and developed using DAB reagent.

### Sample preparation for transmission electron microscopy

Human tissue samples (2-4 mm in diameter) fixed in glutaraldehyde were delivered from the Lung Tissue Research Consortium (LTRC). Upon receipt, they were cut into smaller pieces and re-fixed with one of the following methods: (1) 2% glutaraldehyde in sodium cacodylate containing 0.5% tanic acid or (2) 0.5% reduced osmium in sodium cacodylate. Following overnight fixation, the samples were rinsed in cacodylate buffer. Then, only samples fixed in 2% glutaraldehyde containing 0.5% tanic acid were further fixed in a mixture of 0.75% osmium tetroxide and 0.025M imidazole in 0.1M cacodylate buffer (pH 7.5) for 30 min at 37°C. All fixed samples were then treated with 0.5% uranyl acetate en bloc at room temperature for 2 hrs, followed by dehydration in graded ethanol and acetone. The dehydrated samples were processed for epoxy resin embedding using EMbed-812 (Electron Microscopy Sciences, Hatfield, PA, USA). Thin sections (90-100 nm) were cut using an ultramicrotome (UCT; Reichert). Samples were examined at 80 kV using a transmission electron microscope (1400; JEOL) equipped with a digital camera (Gatan, Inc.).

### Sample preparation for Toluidine Blue staining

The same resin-embedded lung samples as above with the fixation method of glutaraldehyde containing tanic acid followed by a mixture of osmium tetroxide and imidazole were sectioned using an ultramicrotome (UCT; Reichert). Thin sections (350-400 nm) were subjected to Toluidine Blue Staining.

### Western Blotting

Protein extracts from human lung samples were mixed with 4x SDS sample buffer (Boston BioProducts, Worcester, MA) and boiled for 5 min at 95°C. Equal amount of protein (50 μg) were subjected to sodium dodecyl sulfate polyacrylamide gel electrophoresis (SDS-PAGE). Proteins were then transferred onto polyvinylidene difluoride membranes (PVDF). The membranes were blocked in Tris-buffered saline (20 mM Tris·HCl at pH 7.5 and 150 mM NaCl) with 0.1% Tween 20 (TBS-T) containing 5% skim milk, and then incubated with rabbit anti-claudin-10 antibody (Life Technologies, Carlsbad, CA) or rabbit anti-betta-actin antibody conjugated with HRP (Cell Signaling Technology, Beverly, MA). The membranes were washed with TBS-T and claudin-10 antibody treated membrane was further incubated with HRP-conjugated secondary antibody (Jackson ImmunoResearch Laboratory, Inc., West Grove, PA) for 60 min at room temperature. The protein bands were detected on an autoradiography film (MIDSCI #BX57, St. Louis, MO) using Pierce ECL Western blotting substrate (Thermo Fisher Scientific, Hudson, NH).

### Measurement of protein concentration

Protein concentrations of tissue lysates and BAL fluid were determined by BCA assay (cat# 23227; Pierce, Rockford, IL).

### Statistical analysis

Statistical analysis was performed using Graph-Pad Prism 5 (GraphPad Software, San Diego, CA). Comparison of variables between two groups was performed using Student’s t-test. All tests were two-tailed, and values of p < 0.05 were considered significant.

## SUPPLEMENTARY MATERIAL

Supplementary Figure
