# Supplementary material for: Oxidative stress induces club cell proliferation and pulmonary fibrosis in Atp8b1 mutant mice
Source: Aging (Albany NY). 2019 Jan 13;11(1):209–29. doi: 10.18632/aging.101742 (PMC6339797; doi:10.18632/aging.101742)
Supplement: Supplementary Figure [file aging-11-101742-s001.pdf]

SUPPLEMENTARY MATERIAL

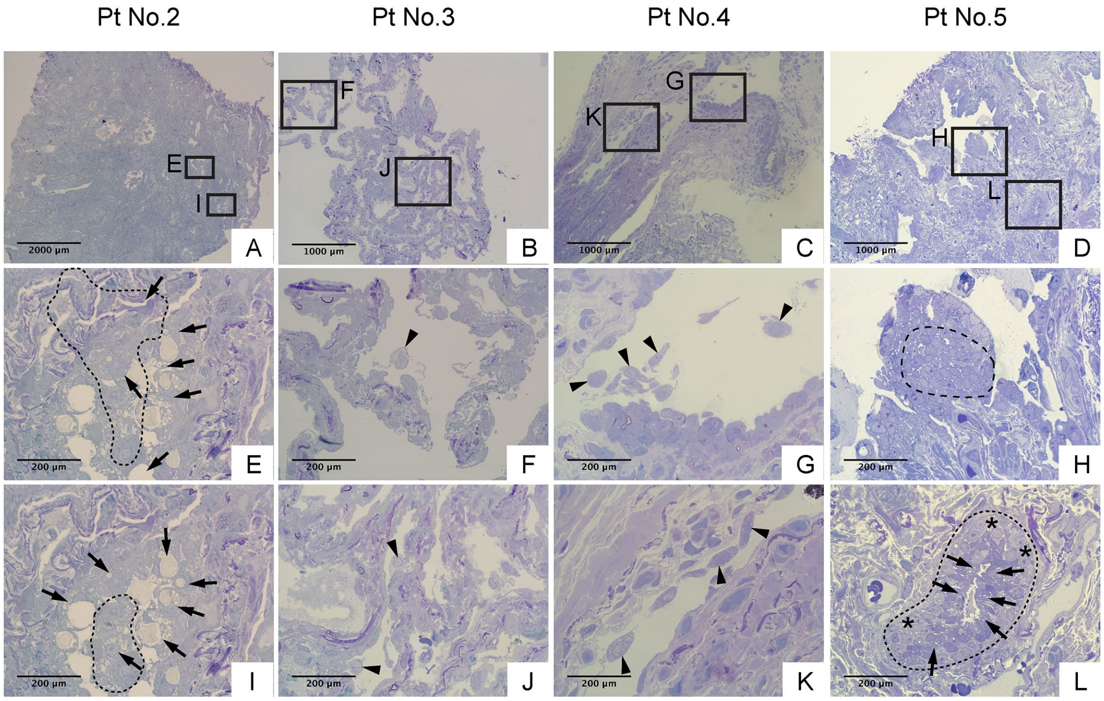

Supplementary Figure 1. IPF lungs display disorganized spreading of morphologically abnormal epithelial cells. Lung samples from IPF patients (n=5) were fixed in glutaraldehyde. Thins slice lung sections (350-400 nm) were prepared and stained with Toluidine Blue. Shown here are representative photomicrographs from four independent patients. Note the lung sections used here in Supp. Fig. 1 and those used for transmission electron microscopy (Fig. 11 & 12) are both from the same tiny lung pieces (a piece 2-4 mm in size each). Labeled boxes correspond to their respective enlarged images. (**A, E & I**) Noted in bronchiolar regions are numerous epithelial cells with cytoplasmic vacuolation (arrows). The airspace is occluded by a clump of cells (areas circled by dashed lines) (**B**) Alveolar region with mild to moderate fibrosis. (**F**) A vacuolated cell is seen in airspace of apparently unaffected alveoli (arrowhead). (**J**) Euchromatic cells are observed in alveoli with mild fibrosis (arrowheads). (**C, G & K**) Cells with varying degrees of cytoplasmic vacuolation are floating in the airspace of relatively large bronchioles. (**D**) Relatively large bronchiole with moderate fibrosis. (**H**) Hyperplastic bronchiolar epithelium featuring distinct cell type with euchromatic nucleus and high N/C ratio (area circled by dashed line). (**L**) Bronchoilar epithelium consisting of two distinct layers of epithelial cell types (area circled by dashed line). Cells with varying degrees of cytoplasmic vacuolation form apical layer (arrows) while euchromatic cells with high N/C ratio (asterisks) are located underneath the apical epithelial layer. Magnifications: (A) 10X; (B, C & D) 20x; (E, F, G, H, I, J, K, L & H) 100X
